# Prevalence and contributory factors to burnout in the New Zealand surgical specialist and registrar population

**DOI:** 10.3389/fpubh.2025.1541892

**Published:** 2025-02-13

**Authors:** Jhanvi Dholakia, Anantha Narayanan, Nicholas Smith

**Affiliations:** ^1^Department of General Surgery, Waikato Hospital, Hamilton, New Zealand; ^2^Department of Vascular Surgery, Waikato Hospital, Hamilton, New Zealand

**Keywords:** surgery, burnout, workforce, stress, McEwan Burnout Questionnaire, wellbeing, resilience

## Abstract

Burnout is a growing phenomenon among medical professionals due to aging patient populations and an increasing burden of chronic disease, in a resource constrained environment. We aim to quantify the prevalence of burnout in surgical specialists and registrars at a tertiary center in New Zealand and identify contributory factors, using a New Zealand based tool, the McEwan Burnout Questionnaire. Of the 110 people surveyed, 55% respondents had concern or high risk of burnout. Contributory factors were frustration with management, lack of resources and long working hours, with predominance toward fatigue and service provision over career progression among the registrar group. Bullying and harassment were reported more in the sub-specialty groups. More time in private practice appeared to be associated with less concern for burnout. These high rates of burnout require targeted interventions toward contributory factors to protect our workers and to maintain a sustainable workforce.

## Introduction

Occupational burnout results from overwhelming unmanaged workplace stress that leads to mental exhaustion, dissociation from one’s work, negative feelings and reduced efficacy (1). In recent years, increasing rates of burnout have been reported in the doctor population as worldwide workforce shortages have exacerbated working conditions, compounded by stressors such as budget cuts, bureaucratic processes and lack of autonomy (2–4). Doctors with burnout describe being unable to empathize with their patients, a sense of depersonalization and a fall in self-worth (5). Surgeons are particularly prone to burnout, highlighted in Medscape survey of twelve thousand American doctors with 45% of the general surgeons experiencing burnout, with similar findings among orthopedic and plastic surgical specialties (6, 7). Attributable factors include poor work-life balance, long working hours, erratic on call schedules, inability to cope with patient suffering, and hospital management issues (8–11). An individual surgeon suffering from burnout is at risk of increased rates of psychiatric comorbidities, alcohol and substance use, and suicide and is also associated with poorer patient outcomes (12–14). There is a greater association of burnout with patient errors, which is associated with higher rates of depression in physicians (3). Patients treated by depersonalized physicians are reported to have longer recovery periods once they leave hospital (15). Burnout leads to higher attrition rates for surgeons, further worsening workforce shortages and leading to increased financial costs to healthcare (16). This study by Han et al. has estimated the annual cost of burnout to be $4.6 billion in the United States alone (16). Understanding the incidence and predictors of burnout among surgeons is therefore paramount to creating meaningful change in clinician wellbeing.

There are no studies that report burnout across all surgical specialists in New Zealand. The aim of this study is to identify the prevalence of burnout in cross-specialty senior medical officers/surgeons (SMO), training registrars (TR) and non-training registrars (NTR), and establish factors that may be associated with burnout, using a survey tool developed in New Zealand for the resident population.

## Materials and methods

This survey based observational cross-sectional study was conducted at a major tertiary hospital in New Zealand in October 2023. The Consensus-Based Checklist for Reporting of Survey Studies (CROSS) was utilized in the design (17). All surgical SMO, TR and NTR (*n* = 239) at our hospital were invited to participate in our study. Respondents were non-incentivized, and responses were anonymized. Respondents were recruited through email contact and surveys disseminated by SurveyMonkey™, with follow up emails sent at 4 and 8 weeks. Incomplete surveys were excluded from the study.

Demographic data collected included age, gender, current specialty, level of training, level of experience and private and public work division for specialists. The McEwan Burnout Questionnaire (MBQ) (Supplementary material), developed for the New Zealand population, assesses the three parts of the burnout syndrome using 20 questions each answered with a Likert scale (18). Cumulative scores were calculated, which allowed individuals to be stratified according to their risk of burnout into broad categories of “doing well” (0–25), or “not doing well” (>25), with further sub-categories of “concern for burnout” (26–39), “high risk of burnout” (40–59) and “burnt out” (60–80) (18). Additionally, respondents identified factors that contributed to their workplace stress.

TR and NTR were grouped as resident medical officers (RMO). Specialties were grouped to three categories: general surgery, orthopedic surgery and subspecialty surgery (which included cardiothoracic, otolaryngology, maxillofacial, neurosurgery, ophthalmology, plastic and burns surgery, urology and vascular surgery).

### Statistics

Summary statistics were calculated for all outcomes of interest. Parametric data between groups was compared using unpaired student’s *t*-test. Ranked non-parametric data were compared using Mann–Whitney U testing. Categorical data were compared using the Pearson Chi squared and Fisher’s Exact test. A multivariate regression analysis was performed to establish associations between variables. Analysis was carried out using SPSS (v29.0). A *p*-value < 0.05 was considered statistically significant.

### Ethics statement

This project was deemed out of scope of the Health and Disability Ethics Committee (HDEC REF: 2024 OOS 21393). All respondents gave informed consent and completed the questionnaire voluntarily.

## Results

Of the 239 people invited to participate, 110 people completed the survey in its entirety (46% response rate). Of these respondents, 24 (22%) were female and 67 (61%) were SMOs. Demographic data is shown in Table 1. Three respondents did not disclose their specialty, and one respondent did not disclose their gender.

**Table 1:** Demographic data.

		Count (%)
Level	RMO	43 (39%)
	SMO	67 (61%)
Specialty	General surgery	33
	Orthopedic	22
	Subspecialty	52
Gender	Female	24 (22%)
	Male	85 (77%)
Age	20–29	9 (8%)
	30–39	46 (42%)
	40–49	37 (34%)
	50–59	13 (12%)
	≥60	5 (4%)

Cumulative scores were calculated from the survey results. The mean burnout score in our cohort was 28 (SD 13.7, range 1–71). As shown in Table 2, in this population 49 people (45%) were doing well, and 61 (55%) were “not doing well.” Of these, 37 (34%) had concern for burnout, 23 (21%) were at high risk of burnout and 1 (1%) person was burnt out. In the RMO group, 65% respondents presented as “not doing well,” compared to 51% in the SMO group (*p* = 0.25). The highest risk age group was 20–29 years with 67% respondents presenting as “not doing well.” There was a non-significant tendency toward lower mean burnout scores as clinician age increased. In the individuals over the age of 60 years, all 13 respondents were “doing well.”

**Table 2:** Burnout by demographic.

		Mean score	Doing well (%)	Not doing well (%)*	*p*
Level	RMO	31	15 (35%)	28 (65%)	
	SMO	27	34 (51%)	33 (49%)	0.1
Specialty	General surgery	27	14 (42%)	19 (58%)	
	Orthopedic	28	12 (54%)	10 (46%)	
	Subspecialty	30	21 (40%)	31 (60%)	0.52
Gender	Female	29	11 (46%)	13 (54%)	
	Male	28	38 (45%)	47 (55%)	0.92
Age	20–29	33	3 (33%)	3 (67%)	
	30–39	29	19 (41%)	27 (59%)	
	40–49	29	16 (43%)	21 (57%)	
	50–59	25	6 (46%)	7 (54%)	
	≥60	13	5 (100%)	0	0.4

*Not doing well includes concern for burnout, high risk of burnout, and burnt out.

In the SMO group, 20 respondents (30%) spent more than one third of their clinical time in private practice. Of this subgroup, only 5% scored as “not doing well” compared to 26% in the group that spent less than one-third of their clinical time in private practice (*p* = 0.05), suggesting that clinical time spent in private practice may be associated with lower burnout rates. Subgroup analysis showed that in the RMO group, 8 (30%) male respondents were “not doing well,” compared to 10 (59%) female respondents (*p* = 0.06). This tendency toward gender difference was not seen in the specialist SMO population. Of the different specialties, cardiothoracic and, plastic and burns surgery presented with the greatest proportion of people “not doing well,” 88 and 62%, respectively. Specialties where the majority of respondents were doing well included neurosurgery (57%), vascular (57%), and orthopedics (55%).

Bullying and harassment contributed to burnout in 21% of respondents in RMO group and 20% of the SMO group. Overall, there were no gender differences in reporting bullying and harassment, however within the RMO subgroup there was a higher reported prevalence among male respondents (25% vs. 4%, *p* = 0.02). Bullying and harassment as a reported contributor to burnout was also noted to be more prevalent in the subspecialty group with 31% compared to General surgery (9%), and Orthopedic surgery (14%) (*p* = 0.037).

Sub analysis by gender showed more females identified acquisition of training positions as a factor contributing to burnout (42%) compared to males (12%) (*p* < 0.001) and there was a non-significant tendency toward females reporting clinical uncertainty to be a contributor of burnout (42%) compared to males (27%) (*p* = 0.08).

Common factors contributing to workplace stress in all groups included frustration with management (66%), lack of resource (64%), long working hours (56%), interference with home life (50%) and fatigue (49%). Factors that did not contribute significantly to burnout included boredom (2%), private practice (7%), and medico-legal concerns (9%). The RMO group compared to the SMO group were more likely to report fatigue (70% vs. 36%, *p* < 0.001) and service provision being valued over career progression (70% vs. 31%, *p* < 0.001) as contributory factors. However, lack of resource was a more common response in the SMO population (76% vs. 47%, *p* = 0.002). The fear of bad outcomes was a contributor of burnout in both SMO (28%) and RMO (44%) groups (*p* = 0.08).

## Discussion

This study demonstrates that burnout has a prevalence of 25% in our surgical population, with a further 33% of doctors showing concern for burnout. This is similar to international literature that shows a burnout rate of 40–80% in surgeons and training surgeons (19–21). Time in private practice appeared to be associated with less concern for burnout in the SMO group. Junior staff were more at risk of burnout and were more likely to report fatigue and service provision as contributory factors. The male RMO and subspecialty groups reported a higher tendency to experience bullying and harassment, while female RMO describe acquisition of training positions and clinical uncertainty to be contributors of burnout.

Our results are in line with published literature highlighting the prevalence of burnout among RMOs. This has been attributed to multiple factors in this group, which include long working hours without breaks, shift work, on-calls, and greater than 80-h work weeks (22–24). These working conditions are known to cause significant physiological strain such as periods of increased heart rate (25) and increased levels of cortisol (26, 27) in junior doctors compared to senior staff associated with on call periods. Multiple programs such as resilience training, mindfulness training, ergonomics workshops and neurofeedback workshops have shown to improve wellbeing and decrease risk of burnout in surgeons and surgical residents (28–30).

Time split between private and public work showed that surgeons who spent more than a third of their time in private practice were at less risk of burnout. This may be related to multiple factors such as increased autonomy, less resource constraints, and less bureaucratic measures in the private sector (31). Private practice also provides greater earning potential which in turn could be associated to improved job satisfaction, a feeling of adequate compensation and therefore, decreases the risk of burnout (32–34).

This study demonstrated a tendency toward female RMOs reporting a higher rate of burnout compared to their male counterparts, which is reflective of prior studies (24, 35). Multiple studies have reported the underlying cause for this gender-based disparity is likely the higher rate of microaggressions by way of discrimination, abuse and harassment that women experience in a surgical workplace (24, 36, 37). A separate study found that women were more likely to receive negative comments about pregnancy from their colleagues, experience more harsh evaluations from their supervisors, and frequently be mistaken for non-medical staff (38). Interestingly, however, in our cohort, males were more likely to report bullying as a cause of burnout than females.

Areas of intervention to decrease burnout can be directed at the individual doctor level, at the hospital level and at a national level. For the doctor, protective factors focus on developing emotional coping strategies to deal with adverse events through resilience training, mindfulness and professional support groups (21, 39). The “Operating with Respect” and “Training in Professional Skills” courses, developed by the Royal Australasian College of Surgeons look to educate individuals on addressing and responding to bullying and harassment and improve conflict resolution skills (40). At the institutional level, mentoring, access to general practitioners, childcare access and support, peer support programs and small discussion groups, and adequate financial compensation are factors which may decrease stressors and thus decrease the rate of burnout (41–43). At the national level recognizing surgeon mental health as collateral damage in an overworked system is important, to then be able to implement safer working hours, increase resources through better staffing, provide more support, and thus decrease attrition thus benefitting the health service (44).

There are limitations to this study. Despite a satisfactory response rate of 46%, respondent bias may skew the results toward those who are interested in or feel strongly toward reporting burnout. The female respondent rate if 22% is slightly lower than the proportion of females in the total surgical workforce (28%) as described by the 2023 Workforce Survey report from the Medical Council of New Zealand, which may influence the gender-based findings in our study. This study was conducted in one major tertiary center with subspecialty departments, the views expressed here may be limited in their generalizability to different tertiary institutions or smaller regional hospitals within or outside of New Zealand.

## Conclusion

In this cohort of 110 surgical specialists and registrars, 55% respondents had concern or high risk of burnout using the McEwan Burnout Questionnaire. Contributory factors were frustration with management, lack of resource and long working hours, with predominance toward fatigue and service provision over career progression among the RMO group. Bullying and harassment were reported more in the sub-specialty groups. More time in private practice appeared to be associated with less concern. These high rates of burnout require targeted interventions toward contributory factors to protect our workers to maintain a sustainable workforce.

## Data Availability

The original contributions presented in the study are included in the article/Supplementary material, further inquiries can be directed to the corresponding author.
