# Supplementary material for: Prevalence and contributory factors to burnout in the New Zealand surgical specialist and registrar population
Source: Front Public Health. 2025 Feb 13;13:1541892. doi: 10.3389/fpubh.2025.1541892 (PMC11865081; doi:10.3389/fpubh.2025.1541892)
Supplement: Supplementary file 1 [file Data_Sheet_1.pdf]

www.drstress.co.nz
JOHN C. McEWAN PhD MNZAC
STRESS, GRIEF, TRAUMA CONSULTANT

BURNOUT QUESTIONNAIRE

FOR EACH OF THE FOLLOWING QUESTIONS ASSIGN A NUMBER FROM 0 - 4, AS TO HOW YOU HAVE BEEN FEELING OVER THE LAST WEEK, FROM TODAY.

0 - NEVER TRUE 1 - ONCE IN A WHILE 2 - SOMETIMES
3 - FREQUENTLY TRUE 4 - ALWAYS TRUE

.... 1. , I feel tired. I feel fatigued rather than energetic.
.... 2. I often feel dissatisfied for no apparent reason.
.... 3. I feel sad for no apparent reason.
.... 4. I am forgetful(short term memory fails me at times).
.... 5. I am irritable and bite, snap and snarl at people.
.... 6. I am withdrawn from others.
.... 7. I am having trouble sleeping through the night, <u>or</u> am sleeping more but not feeling refreshed.
.... 8. I am getting sick a lot with "little" or "big" complaints.
.... 9. My attitude towards work is "who gives a toss!".
.... 10. I get into conflicts with others at work and/or home.
.... 11. I am aware that my job performance is not up to par.
.... 12. I use alcohol and or drugs to feel better.
.... 13. Communicating with others is a strain/tiresome.
.... 14. I can't concentrate on tasks as I used to.
.... 15. I am easily bored, yet cannot decide what to do.
.... 16. I am working harder, or longer hours, but accomplishing less.
.... 17 I feel frustrated with things in my life.
.... 18. I find it hard getting up out of bed and going to work.
.... 19. Social activities are energy draining, or have lost their old appeal.
.... 20. Sex is not worth the effort.

------ TOTAL UP SCORE

00 - 25 You are doing well 26 - 39 Some things to watch
40 - 59 You are getting close 60 - 80 Burnout territory

This questionnaire is designed to be used on a three monthly basis to ensure that "Burnout" is not creeping up on you.

Revised 22 January 2003 Dr John C McEwan

130 Wheturangi Road, Epsom, Auckland. Ph: (09) 522 6723, Fax: (09) 522 6724 john.mcewan@xtra.co.nz
